# The Genetic Variants in the Renin-Angiotensin System and the Risk of Heart Failure in Polish Patients

**DOI:** 10.3390/genes13071257

**Published:** 2022-07-15

**Authors:** Iwona Gorący, Anna Gorący, Mariusz Kaczmarczyk, Jakub Rosik, Klaudyna Lewandowska, Andrzej Ciechanowicz

**Affiliations:** 1 Department of Clinical and Molecular Biochemistry, Pomeranian Medical University, 70-111 Szczecin, Poland; iwona.goracy@pum.edu.pl (I.G.); ania.goracy@gmail.com (A.G.); mariusz.kaczmarczyk@pum.edu.pl (M.K.); jakubrosikjr@gmail.com (J.R.); klaudyna.lewandowska@pum.edu.pl (K.L.); 2 Department of Chemistry, The University of Chicago, Chicago, IL 60637, USA

**Keywords:** heart failure, renin-angiotensin system, genetic polymorphism

## Abstract

(1) Background: Heart failure (HF) is a complex disease and one of the major causes of morbidity and mortality in the world. The renin-angiotensin system (RAS) may contribute to the pathogenesis of HF. (2) Aim: To investigate the association of RAS key genetic variants, rs5051 (A-6G) in the gene encoding angiotensinogen (*AGT*), rs4646994 (I/D) in the gene for angiotensin I converting enzyme (*ACE*), and rs5186 (A1166C) in the gene encoding type 1 receptor for angiotensin II (*AGTR1*), with the HF risk in the cohort of Polish patients. (3) Methods: The study group consisted of 415 patients that were diagnosed with HF, while the control group comprised of 152 healthy individuals. Genomic DNA were extracted from blood and genotyping was carried out using either PCR or PCR-RFLP for *ACE* or *AGT* and *AGTR1* variants, respectively. (4) Results: No association has been found between the I/D *ACE* and heart failure. The HF risk was significantly higher for AG *AGT* heterozygotes (overdominance: AG versus AA + GG) and for carriers of the G *AGT* allele in codominant and dominant modes of inheritance. However, the risk of HF was significantly lower in the carriers of at least one C *AGTR1* allele (AC or CC genotypes) or in AC *AGTR1* heterozygotes (overdominant mode). There was a significant relationship for *AGT* and HF patients in NYHA Class I-II for whom the risk was higher for the carriers of the G allele, and for the AG heterozygotes. There was also a significant interaction between heterozygote advantage of *AGT* and BMI increasing the risk for HF. (5) Conclusion: Our results suggest that the A(-6)G *AGT* polymorphism may be associated with HF in the Polish population and the HF risk seems to be modulated by the A1166C *AGTR1* polymorphism.

## 1. Introduction

Heart failure (HF) is now a very important health concern worldwide and despite better medical care and treatment due to an aging society, the incidence of HF will continue to increase [1]. The major causes of HF are coronary artery disease (CAD), arterial hypertension (HT), heart valve disease (VD), or cardiomyopathies. The development of HF is also influenced by many factors, such as neurohormonal, behavioral, and environmental factors. Among these factors, one of the most important roles in the pathophysiology of HF is the renin-angiotensin system (RAS) [2].

The RAS is one of the key players in volume homeostasis and has powerful effects on the heart and circulatory systems. In patients with HF, the relative increase in the activity of the renin-angiotensin system is considered a marker contributing to hemodynamic disturbances [3]. Moreover, the RAS plays an important role in the pathophysiology of left ventricular dysfunction, such as structural (remodeling) changes in the heart and left ventricular hypertrophy (LVH) [4,5]. However, the risk factors of HF are not fully recognized yet, and the genetic determinants of cardiac function are probable contributors. There are several reports that genetic variations in the RAS play important roles in the development of CAD, HT, LVH, or cardiomyopathy [6,7]. The key components of the RAS are angiotensinogen (AGT), angiotensin converting enzyme (ACE), and angiotensin II Type 1 receptor (AGTR1). It has been demonstrated that genetic variation in the *AGT* gene is linked to susceptibility to CAD, HT, or atrial fibrillation [8,9]. The most extensively studied insertion/deletion (I/D) polymorphism of the *ACE* gene has been shown to be associated with increased left ventricular mass or LVH, HT, and CAD [10,11]. Also, for the *AGTR1* gene, a correlation with HT, CAD, and atherosclerosis was shown [12]. On the other hand, although research on the genetic determinants of HF is widely studied, reports showing the relationship of HF still remain controversial.

So far, a possible association of HF with genetic polymorphisms such as the renin-angiotensin-aldosterone system, sympathetic nervous system, or inflammatory genes [13], has been demonstrated. However, there are many reports in various populations that do not support this relationship [13]. However, extensive research continues to look for genetic factors influencing the development of HF that would aid in a better understanding of the mechanisms that lead to the development or progression of HF.

In the present study, we investigated the association between A(-6)G *AGT* (rs5051), I/D *ACE* (rs4646994), and A1166C *AGTR1* (rs5186) polymorphisms and HF in a Polish population.

## 2. Patients and Methods

### 2.1. Patients

This was a retrospective study of patients that were diagnosed with chronic HF referred to the Department of Cardiac Surgery, Pomeranian Medical University, for treatment. The study group was from a homogeneous Polish population. A total of 415 patients (306 men and 109 women) were included in the study. The study group with HF included patients with coronary artery disease (CAD) (n = 254, 201 men and 53 women), patients with valvular disease (VD) (n = 68, 31 men and 37 women), and patients with combined disease: ischemic heart disease + valvular disease (CD) (n = 93, 68 men and 25 women). The study was comprised of patients with chronic HF of the New York Heart Association (NYHA) functional Class I–IV. The NYHA Class I included 53 patients, Class II included 215 patients, Class III included 130 patients, and Class IV included 17 patients. The study population was divided according to the NYHA classification according to the severity of the disease: group one, patients with NYHA Classes I and II, and group two, patients with NYHA Classes III and IV. The demographic data and medical history of patients were collected from their medical records.

The control group consisted of 152 volunteers (107 men and 45 women) in whom a medical examination ruled out HF and other cardiovascular diseases. All the study participants underwent transthoracic echocardiography. The protocol of the study was approved by the Pomeranian Medical University Ethics Committee with formal informed consent signed by all the participants.

### 2.2. Genotyping

Genomic DNA were extracted from peripheral blood leukocytes with the QIAamp DNA Mini Kit (Quiagen, Hilden, Germany). Polymerase chain reaction-restriction fragment length polymorphism (PCR-RFLP) and polymerase chain reaction (PCR) were used to identify the polymorphisms of the selected genes. Genotyping of the G(-6)A *AGT* (rs5051), I/D *ACE* (rs4646994), and A1166C *AGTR1* (rs5186) polymorphisms was carried out according to the previously described method [14].

### 2.3. Statistical Analysis

Hardy–Weinberg equilibrium was analyzed using the chi-square test. Associations analysis was performed using a generalized linear model (GLM) in which a predicted probability of a response variable was modeled with a binomial distribution and a logit link function. A GLM was used in a single marker analysis, and to model pairwise gene-gene interactions as well as gene–environmental interactions. In single marker analysis, four genetic models were considered, i.e., codominant (coded as two indicator variables), dominant (genotypes with allele of interest share code 1), recessive (homozygous genotyped coded as 1), and overdominant (heterozygous advantage, heterozygous genotype coded as 1). A total of six types of gene–gene interactions were specified according to the number and types of alleles in the two interacting genotypes, i.e., dominant — the presence of at least one allele of interest, recessive — two homozygous genotypes, homozygote-heterozygote model — homozygous genotype in the first locus and heterozygous genotype in the second locus (HOM-HET model), and heterozygote-homozygote model—heterozygous genotype in the first locus and homozygous genotype in the first locus (HET-HOM model). Where indicated, p values were adjusted for multiple comparisons using the Benjamini and Hochberg procedure to control for the false discovery rate (FDR). The significance of genetic components and gene–environmental terms of the models was tested using likelihood ratio test by comparing a full and reduced models. All analyses were performed using R (https://cran.r-project.org/, accessed on 1 of June 2022).

## 3. Results

The demographic characteristics of the HF and control groups are shown in Table 1. BMI and age proved to be significantly higher in the HF group when compared to the control group, and male gender was significantly more frequent. Similarly, smoking, diabetes, and HT were more common among the HF group when compared to the control group (Table 1).

### 3.1. Association between Overall HF and Genetic Variation in AGT, ACE, and AGTR1

The genotype distributions in the control group were in Hardy–Weinberg equilibrium (*p* = 0.516, *p* = 1.0, and *p* = 0.210 for the I/D *ACE*, A1166C *AGTR1,* and A(-6)G *AGT*, respectively). The results of tests for association of the A(-6)G *AGT*, I/D *ACE,* and A1166C *AGTR1* polymorphisms with HF are summarized in Table 2, Table 3 and Table 4. For the A(-6)G *AGT* gene polymorphism, the association under the codominant, dominant, and overdominant models was significant after adjustment for covariates (age, sex, BMI, smoking, diabetes mellitus, and hypertension). For the codominant model, the risk of HF was 3.15-fold higher (95% CI 1.4–7.05, *p* = 0.015) for the G/A heterozygotes (but not for G/G homozygotes) compared with A/A homozygotes. Also, for the dominant model, the risk of HF for the carriers of the G allele was 2.62-fold higher (95% CI 1.26–5.51, *p* = 0.010) compared with the A/A homozygotes, and for the overdominant model, HF in G/A heterozygotes occurred more frequently than in A/A–G/G homozygotes, which conferred a 2.11-fold higher risk of HF (95% CI 1.16–3.95, *p* = 0.015) compared with both homozygotes (Table 2). The results of tests for association of the I/D *ACE* polymorphism with HF showed no associations with this marker (Table 2). For the A1066C *AGTR1* polymorphism under the dominant model, the carriers of the C allele (A/C-C/C) had a reduced risk of HF (OR = 0.54, 95% CI 0.029–0.97, *p* = 0.039), and finally for the overdominant model, A/C heterozygotes occurred less frequently than A/A–C/C homozygotes, which conferred a lower risk of HF (OR = 0.47, 95% CI 0.25–0.87, *p* = 0.016) (Table 2). Table 3 shows the analysis of gene–gene interaction with respect to the risk of HF; no significant relationship was found in this association.

In addition to the analysis of single sites, we examined whether the three genes that were studied affect the modification of risk factors (BMI and HT) of HF. We recorded a significant interaction only for the overdominant model for AGT and BMI increasing the risk for HF (*p* = 0.038). For HT, we did not find any significant gene–environmental interactions (Table 4).

### 3.2. Association between HF in NYHA Class I/II and III/IV and Genetic Variation in AGT, ACE, and AGTR1

We repeated an analysis in a subset of patients that were classified according to the NYHA classification. The results of tests for association of the *AGT*, *ACE,* and *AGTR1* polymorphisms with the HF group with NYHA Class I/II are summarized in Supplementary Table S1. For the G(-6)A *AGT* gene polymorphism, the association under the codominant, dominant, and overdominant models was significant after adjustment for covariates (age, sex, BMI, smoking, diabetes mellitus, and hypertension). For the codominant model, the risk of HF was 4.07 times higher (95% CI 1.63–10.5, *p* = 0.010) for the G/A heterozygotes and 2.91 times higher (95% CI 1.12–7.78, *p* = 0.010) for the G/G homozygotes compared with A/A homozygotes. For the dominant model, the risk of HF in the group with NYHA Class I/II for the carriers of the allele G was 3.53 times higher compared with the A/A homozygotes (95% CI 1.51–8.50, *p* = 0.004), and for the overdominant model, G/A heterozygotes occurred more frequently than A/A–G/G homozygotes, conferring a higher risk of HF (OR = 2.05, 95% CI 1.05–4.13, *p* = 0.036) compared with both homozygotes (Supplementary Table S1). No associations were found for the I/D *ACE* gene polymorphism in those with NYHA Class I/II (Supplementary Table S1). For the A1066C *AGTR1* gene polymorphism under the codominant model, the risk of HF in the group with NYHA Class I/II was reduced 2.5-fold among the A/C heterozygotes compared with the A/A homozygotes (OR = 0.40, 95% CI 0.19–0.80, *p* = 0.021). For the dominant model, the carriers of the C allele (A/C–C/C) had a 2.1-fold reduction of the risk for HF (OR = 0.48, 95% CI 0.24–0.92, *p* = 0.027). In the overdominant model, HF occurred less frequently in the A/C heterozygotes than in the A/A–C/C homozygotes, which conferred a 2.63-fold lower risk of HF (OR = 0.38, 95% CI 0.19–0.76, *p* = 0.006) (Supplementary Table S1). The gene–gene interactions with the risk in a group of patients with NYHA Class I/II HF were also studied (Supplementary Table S2). In the dominant and recessive models for all the pairwise interactions, we noted no significant association. However, for the *ACE* and *AGT* pair, we found an interaction under the HET-HOM2 model. The presence of the heterozygous ID genotype of the *ACE* gene and the homozygous AA genotype of the *AGTR1* gene reduced the risk of HF 2.43-fold (OR = 0.14, 95% CI 0.04–0.52, *p* = 0.018). For the *AGTR1* and *AGT* pair for the HOM2-HET model (the presence of the homozygous AA genotype of the *AGTR1* gene and the heterozygous AG genotype of the *AGT* gene), we have demonstrated a 2.82-fold higher risk of HF (OR = 2.82, 95% CI 1.32–6.26, *p* = 0.042). For the other gene-gene models, the associations were insignificant.

No significant associations were found between the G(-6)A*AGT*, I/D *ACE* and A1166C *AGTR1* polymorphisms and severe HF in those with NYHA Class III/IV under the codominant, dominant, and recessive models (Supplementary Table S3). Also, in the gene–gene interaction for the risk of NYHA Class III/IV HF, we noted no significant association (Supplementary Table S4).

## 4. Discussion

Although the association between the RAS and cardiovascular diseases has been demonstrated in many studies, the association of the RAS system with HF is still controversial. Our study reported an increased risk of HF for the G(-6)A *AGT* gene polymorphism (under the codominant, dominant, and overdominant models). Our results suggest that the AG genotype was prevalent in patients with HF, which may suggest an association of heterozygotes with a higher risk of HF in our population. Only in the dominant model, a higher risk of HF was associated with the carriers of the G allele in our population. Moreover, we showed that the A1166C polymorphism of the *AGTR1* gene may have a modulating effect on HF (under dominant and overdominant models), whereas for the AC heterozygotes, we noted a lower risk of HF for the Polish population. We have not demonstrated the relationship between the I/D *ACE* polymorphism and HF in Polish patients.

The classical angiotensinogen-angiotensin I-angiotensin II pathway promotes vasoconstriction, inflammation, oxidative stress, cell proliferation, and cardiomyocyte hypertrophy by stimulating the Type 1 receptor for angiotensin II [15]. Angiotensinogen, as an initial step in this pathway, may be of crucial importance, as any variability that causes its serum concentration to increase can be considered deleterious. The most frequently studied polymorphism of M235T *AGT* (rs699) so far shows that the concentration of angiotensinogen in the serum is increased in a stepwise manner with the number of T235 alleles [16,17]. In our study, we examined the relationship between the A(-6)G *AGT* polymorphism and HF. This polymorphism remains in a very tight linkage disequilibrium with the M235T *AGT* substitution, and it is believed that the A(-6)G variation affects the basal rate of *AGT* gene transcription [18]. Elevation of circulating AGT levels are associated with an increase in the concentration of angiotensin II, which activates cardiomyocyte hypertrophy and fibroblast proliferation by stimulating the AT1 receptor. Furthermore, it activates vascular and myocardial cell apoptosis and contributes to cardiac remodeling [19,20]. The important role of AGT in regulating the RAS is that AGT may likely modulate the risk of HF. Therefore, the *AGT* polymorphism has been widely researched as an HF candidate gene. Many studies show an association between the M235T *AGT* polymorphism and HF, which is consistent with our results. Zakrzewski et al. [21] showed an association of the 235T allele with HF by examining a small group of Canadian Caucasians. In the Tunisian population, a high risk of HF, as well as a high risk of death, was found in TT homozygotes of the *AGT* gene [22]. In a Spanish study, the authors demonstrated that the T allele of *AGT* is responsible for CAD and may be involved in increasing the risk of HF in these patients [23]. Also, Goldbergova et al. investigated the relationship of M235T and A(–6)G of *AGT* polymorphisms in 158 patients from a Czech population with chronic heart failure (CHF). In this study, they provided evidence for an increased risk in people with GG-MT of the *AGT*-related genotype variant for CHF, especially a 15-fold risk of this variant in women [24], whereas most studies did not find an association between genetic variants of *AGT* and the development of HF [25]. However, it should be emphasized that the relationship of *AGT* genetic variants may differ ethnically. In one meta-analysis, it was indicated that the M235T polymorphism may be associated with HF risk in Caucasians [26], while another meta-analysis indicated that the M235T *AGT* polymorphism was a low-penetrant risk factor for the development of HF among Asians, which could lead to reducing the risk of HF among Asians [27]. This suggests a possible role of ethnic differences in genetic origins and in different environmental influences. It is important to underline the fact that our results stem from a representative cohort of a homogeneous Polish population of individuals with HF.

The most studied genetic variant of the RAS are polymorphisms of the A1166C *AGTR1* and the I/D *ACE* in the files of cardiovascular diseases. ACE is a key enzyme catalyzing the production of angiotensin II, and the AGTR1 is the major receptor of angiotensin II, which mediates most of the physiologic actions of angiotensin II. Although A1166C of the *AGTR1* gene polymorphism does not appear to be functional, it tends to be in a linkage disequilibrium with unidentified functional loci, which would affect the regulation of the gene. Earlier, the report confirmed that the A1166C polymorphism is located in the miRNA binding sites, and miR155 binding at its target sites reduces the expression of only 1166A not the 1166C allele of the *AGTR1* gene [28].

The previous findings correlating the A1166C *AGTR1* gene polymorphism with cardiovascular diseases are contradicting. Some studies support that the 1166C *AGTR1* allele is a predisposing genetic marker for CAD or MI, while the AA genotype was protective [29,30,31]. There are other reports that are contrary to these findings [32,33]. In the current study, we noted, for the A1166C *AGTR1* polymorphism, that the presence of the AC heterozygote may reduce the risk of HF (after adjusted for age, gender, BMI, smoking, DM, and hypertension). It is worth noting that the increased frequency of the AC heterozygote in the control group may be a protective feature and may be associated with a lower risk for HF, which may be due to the presence of the A allele. Our results are in line with some studies. In a study of Turkish patients with premature coronary heart disease and controls, a significant relationship was found between the 1166AA *AGTR1* genotype and reduced risk of premature CAD [34]. Similarly, among north Indian patients with ID + DD *ACE* and 1166AC + CC *AGTR1*, the combined genotype had a much higher risk of acute myocardial infarction than with II *ACE* and the 1166AA *AGTR1* combined genotype [35]. Furthermore, the meta-analysis showed that the C allele and AC genotype of *AGTR1* was associated with an increased risk of essential hypertension, while a decreased risk of essential hypertension was observed in the A allele and AA genotype [36]. However, it is worth noting that, in the Swedish population, it was reported that the interaction between the AC/CC *AGTR1* and DD *ACE* genotypes is a predictor of survival for patients with HF [37]. Also, Barczewski et al. [21], researching patients with HF concluded that the HF group had a lower proportion of patients with the CC *AGTR1* genotype when compared with healthy controls; however, this result was statistically insignificant. Our previous studies have shown a lack of association between the A1166C *AGTR1* and I/D *ACE* polymorphisms and LVH [38] and MI [39] in the Polish population.

It is known that HF is a complex disease. There are many factors contributing to this problem, including ethnicity, lifestyle, environmental influences, and others that may modulate these dependencies, and gene–gene or gene–environment interaction is still difficult to interpret. Fave et al. [40] demonstrated the direct influence of the local environment on disease risk phenotypes, and genetic variation, including less common variants, can also modulate individual responses to environmental challenges. It should be noted that, in our Polish population, traditional risk factors (e.g., HT, DM, dyslipidemia, smoking, and obesity) are widespread.

We also considered the influence of the A(-6)G*AGT*, I/D *ACE,* and A1166C *AGTR1* polymorphisms on HF depending on the severity of the disease according to the NYHA classification (data in Supplementary Material). In the group with NYHA Class I/II, for the *AGT* genetic variants, our results suggested that the AG heterozygotes had an increased risk of HF under the codominant and overdominat models, as well as the carriers of the G allele under the dominant model. Whereas, for the A1166C *AGTR1* polymorphism in the NYHA Class I/II group, we noted a significant relationship in the codominant, dominant, and overdominant models. In this relationship, our results indicated a similar occurrence in the whole group—a lower risk of HF for AC heterozygotes. For HF patients with NYHA Class III/IV, we noted a lack of associations with the G(-6)A *AGT*, I/D *ACE,* and A1166 *AGTR1* polymorphisms. The obtained results may mean that, in our cohort of patients with HF, the influence of genetic factors is weak, and it may be due to the strong influence of traditional risk factors and environmental factors. This fact may be proved by the weakly marked influence of the studied genes among patients with HF in NYHA Class III/II, which disappears completely among patients with advanced HF in NYHA Class III/IV. In the NYHA Class III/IV group, the risk factors for HF (e.g., myocardial hypertrophy and HT) may have a much stronger influence than genetic factors due to the duration of the disease.

Moreover, the obtained results of the *AGT*-BMI interaction are also interesting, which indicates that the A(-6)G AGT polymorphism plays a significant role in the overdominant model in the Polish population. Obesity and being overweight are known to be important factors in the development and progression of HF. In our study population, the prevalence of overweight/obesity is strongly expressed, and the modulating effect of the *AGT* genetic variants may be consistent with this feature. Obesity is a complex quantitative trait that is considered to be influenced by both genetic and environmental factors. The *AGT* gene has been shown to play a role in regulating the growth and differentiation of adipose tissue [41]. In earlier studies, Umemura et al. [42] showed that obesity, plasma AGT levels, and blood pressure are positively related to BMI. Also, Giacchetti et al. reported a correlation between angiotensinogen expression in the adipose tissue of obese patients and BMI in visceral adipose tissue [43]. Vasku et al. [44], in their study, presented that subjects with the A allele of the A(-6)G *AGT* polymorphism were prone to gain greater body weight than G/G homozygotes during 3-years of observation without strong intervention of a diet. On the other hand, Takakura at al. [45] demonstrated that the T/T genotype of the M235T *AGT* gene polymorphism is linked to visceral obesity and insulin resistance in obese Japanese women, and this genotype is regarded to have greater risk for obesity-related diseases in obese Japanese women. However, the data in the literature are contradictory, and some studies were not able to find associations between BMI and *AGT* variants [46,47]. No significant interactions have been found between RAS genetic variants and hypertension. However, it is worthy to note that the prevalence of hypertension in our HF group was very high (almost 85%). On the other side, Rodriguez-Perez et al. revealed that the M235T *AGT* polymorphism, which is in very tight linkage disequilibrium with the A(-6)G *AGT* transition, contributes to the presence of coronary artery disease independently of the blood pressure profile [23]. The limitation of this study was the relatively small size of both groups. It should also be emphasized that the etiology of HF in our patients was diverse. In addition, the controls were significantly younger than the HF subjects. There were also significant differences between both groups in the BMI value and in the prevalence of smoking, diabetes mellitus, and hypertension. Therefore, the likelihood ratio test adjusted for age, sex, BMI, smoking, diabetes mellitus, and hypertension was performed to verify whether the associations of RAS genetic polymorphisms with the HF risk are independent of these factors.

## 5. Conclusions

The results of our study confirm the relationship between *AGT* genotypes and an increased risk for HF in a Polish population, while *AGTR1* genotypes are associated with a reduction in risk for HF. HF is a complex disease—many environmental risks factors play important roles in its development, but genetic factors may partially predispose or modulate to varying degrees in different populations and geographic locations. However, further studies should be carried out in order to help determine the influence of genes, gene–gene interactions or gene–environmental interactions in various populations.

## Figures and Tables

**Table 1:** Basic characteristics of the HF patients and control subjects.

Variable	HF Patients (n = 415)	Control Subjects (n = 152)	*p* *
Males, n (%)	306 (73.7%)	107 (70.4%)	0.493
Age, years	66.3 ± 8.4	53.9 ± 9.3	<0.001
BMI, kg/m^2^	29.0 ± 4.5	27.4 ± 3.6	<0.001
Smoking, n (%)	134 (32.3%)	32 (21.1%)	0.012
Diabetes mellitus, n (%)	146 (35.2%)	9 (5.9%)	<0.001
Hypertension, n (%)	352 (84.8%)	24 (15.8%)	<0.001

* *t*-test or Chi-square test.

**Table 2:** Association of the A(-6)G *AGT*, I/D *ACE,* and A1166C *AGTR1* polymorphisms with heart failure in regard to codominant, dominant, recessive, or overdominant model of inheritance.

Gene	Genotypes	HF Patients (n = 415)	Controls (n = 125)	OR (95% CI)	*p* *
*AGT*	Codominant model				
	AA	80 (19.3)	37 (29.6)	1	0.015
	AG	219 (52.8)	55 (44.0)	3.15 (1.43–7.05)	
	GG	116 (28.0)	33 (26.4)	1.96 (0.84–4.61)	
	Dominant model				
	AA	80 (19.3)	37 (29.6)	1	0.010
	AG + GG	335 (80.7)	88 (70.4)	2.62 (1.26–5.51)	
	Recessive model				
	AA + AG	299 (72.0)	92 (73.6)	1	0.697
	GG	116 (28.0)	33 (26.4)	0.88 (0.46–1.69)	
	Overdominant model				
	AA+GG	196 (47.2)	70 (56.0)	1	0.015
	AG	219 (52.8)	55 (44.0)	2.11 (1.16–3.95)	
*ACE*	Codominant model				
	II	97 (23.4)	36 (23.7)	1	0.218
	ID	213 (51.3)	81 (53.3)	0.65 (0.30–1.36)	
	DD	105 (25.3)	35 (23.0)	0.46 (0.19–1.10)	
	Dominant model				
	II	97 (23.4)	36 (23.7)	1	0.139
	ID + DD	318 (76.6)	116 (76.3)	0.59 (0.28–1.19)	
	Recessive model				
	II + ID	310 (74.7)	117 (77.0)	1	0.187
	DD	105 (25.3)	35 (23.0)	0.63 (0.32–1.25)	
	Overdominant model				
	II + DD	202 (48.7)	71 (46.7)	1	0.912
	ID	213 (51.3)	81 (53.3)	0.97 (0.54–1.74)	
*AGTR1*	Codominant model				
	AA	225 (54.2)	80 (52.6)	1	0.055
	AC	161 (38.8)	61 (40.1)	0.48 (0.25–0.89)	
	CC	29 (6.7)	11 (7.2)	1.03 (0.32–3.50)	
	Dominant model				
	AA	225 (54.2)	80 (52.6)	1	0.039
	AC + CC	190 (45.8)	72 (47.4)	0.54 (0.29–0.97)	
	Recessive model				
	AA + AC	386 (93.0)	141 (92.8)	1	0.577
	CC	29 (7.0)	11 (7.2)	1.39 (0.45–4.54)	
	Overdominant model				
	AA + CC	254 (61.2)	91 (59.9)	1	0.016
	AC	161 (38.8)	61 (40.1)	0.47 (0.25–0.87)	

* Statistical significance tested by the likelihood ratio test, adjusted for age, sex, BMI, smoking, diabetes mellitus, and hypertension.

**Table 3:** Pairwise gene variants interactions and the risk of HF assuming different models.

rs 1	rs 2	Model OR (95% CI), *p* *					
		DOM	REC	HOM1-HET	HOM2-HET	HET-HOM1	HET-HOM2
*ACE*	*AGTR1*	0.46 (0.24–0.83) 0.060	0.98 (0.13–8.95) 0.987	DD-AC 0.65 (0.23–1.91) 0.511	II-AC 1.76 (0.60–5.38) 0.462	ID-CC 2.46 (0.529–1.31) 0.462	ID-AA 2.09 (1.07–4.21) 0.090
*ACE*	*AGT*	1.32 (0.72–2.43) 0.472	0.58 (0.19–1.93) 0.472	DD-AG 1.08 (0.44–2.79) 0.867	II-AG 1.90 (0.65–5.69) 0.472	ID-GG 0.70 (0.31–1.61) 0.472	ID-AA 0.35 (0.13–0.98) 0.276
*AGTR1*	*AGT*	0.94 (0.50–1.74) 0.834	5.89 (0.23–387.9) 0.443	CC-AG 4.09 (0.83–24.5) 0.168	AA-AG 2.35 (1.19–4.76) 0.078	AC-GG 0.56 (0.24–1.32) 0.275	AC-AA 0.30 (0.09–1.11) 0.168

* FDR-adjusted *p* values for each pair of gene variants separately.

**Table 4:** The interaction of genetic variants with BMI or hypertension and the risk of HF assuming different models.

Gene	Codominant Model	Dominant Model	Recessive Model	Overdominant Model
	BMI			
*ACE*	Chisq = 1.36 (0.508)	Chisq = 0.05 (0.828)	Chisq = 1.33 (0.249)	Chisq = 4.85 (0.089)
*AGTR1*	Chisq = 0.52 (0.772)	Chisq = 0.44 (0.509)	Chisq = 0.002 (0.963)	Chisq = 0.51 (0.476)
*AGT*	Chisq = 4.44 (0.109)	Chisq = 0.26 (0.607)	Chisq = 2.93 (0.087)	Chisq = 4.31 (0.038)
	Hypertension			
*ACE*	Chisq = 0.29 (0.865)	Chisq = 0.26 (0.613)	Chisq = 0.10 (0.750)	Chisq = 2.64 (0.267)
*AGTR1*	Chisq = 0.12 (0.941)	Chisq = 0.09 (0.764)	Chisq = 0.10 (0.757)	Chisq = 0.001 (0.975)
*AGT*	Chisq = 3.40 (0.182)	Chisq = 0.002 (0.958)	Chisq = 3.11 (0.078)	Chisq = 1.89 (0.169)

## Data Availability

The data presented in this study are available on request from the corresponding author.

## Supplementary Materials

The following supporting information can be downloaded at: https://www.mdpi.com/article/10.3390/genes13071257/s1, Table S1 Association of the A(-6)G AGT, I/D ACE, and A1166C AGTR1 polymorphisms with heart failure in NYHA Class I-II patients in regard to codominant, dominant, recessive, or overdominant model of inheritance; Table S2 Pairwise gene variants interactions and the risk of HF of NYHA Class I/II assuming different models; Table S3 Association of the A(-6)G AGT, I/D ACE, and A1166C AGTR1 polymorphisms with heart failure in NYHA Class III-IV patients in regard to codominant, dominant, recessive or overdominant, model of inheritance; Table S4 Pairwise gene variants interactions and the risk of HF of NYHA Class III/IV assuming different models.
