# Supplementary material for: The Genetic Variants in the Renin-Angiotensin System and the Risk of Heart Failure in Polish Patients
Source: Genes (Basel). 2022 Jul 15;13(7):1257. doi: 10.3390/genes13071257 (PMC9319667; doi:10.3390/genes13071257)
Supplement: Supplementary file 1 [file genes-13-01257-s001.zip › genes-1796013-supplementary.pdf]

Supplementary materials

The Genetic Variants in the Renin-Angiotensin System and the Risk of Heart Failure in Polish Patients

Iwona Gorący [1], Anna Gorący [1], Mariusz Kaczmarczyk [1], Jakub Rosik [1,2], Klaudyna Lewandowska [1] and Andrzej Ciechanowicz [1,*]

Supplementary Table S1. Association of the A(-6)G *AGT*, I/D *ACE* and A1166C *AGTR1* polymorphisms with heart failure in NYHA class I-II patients in regard to codominant, dominant, recessive or overdominant model of inheritance.

Gene	Genotypes	HF patients	Controls	OR (95% CI)	p*
			Codominant model		
	AA	43 (16.0)	37 (29.6)	1	0.010
	AG	146 (54.5)	55 (44.0)	4.07 (1.63-10.5)	
	GG	79 (29.5)	33 (26.4)	2.91 (1.12-7.78)	
			Dominant model		
	AA	43 (16.0)	37 (29.6)	1	0.004
AGT	AG+GG	225 (84.0)	88 (70.4)	3.53 (1.51-8.50)	
			Recessive model		
	AA+AG	189 (70.5)	92 (73.6)	1	0.846
	GG	79 (29.5)	33 (26.4)	1.07 (0.53-2.19)	
			Overdominant model		
	AA+GG	122 (45.5)	70 (56.0)	1	0.036
	AG	146 (54.5)	55 (44.0)	2.05 (1.05-4.13)	
			Codominant model		
	II	73 (27.2)	36 (23.7)	1	0.141
	ID	129 (48.1)	81 (53.3)	0.49 (0.21-1.10)	
	DD	66 (24.6)	35 (23.0)	0.41 (0.15-1.07)	
			Dominant model		
	II	73 (27.2)	36 (23.7)	1	0.053
ACE	ID+DD	195 (72.8)	116 (76.3)	0.46 (0.20-1.01)	
			Recessive model		
	II+ID	202 (75.4)	117 (77.0)	1	0.332
	DD	66 (24.6)	35 (23.0)	0.69 (0.32-1.46)	
			Overdominant model		
	II+DD	139 (51.9)	71 (46.7)	1	0.428
	ID	129 (48.1)	81 (53.3)	0.77 (0.40-1.47)	
			Codominant model		
	AA	149 (55.6)	80 (52.6)	1	0.021
	AC	99 (36.9)	61 (40.1)	0.40 (0.19-0.80)	
	CC	20 (7.5)	11 (7.2)	1.31 (0.34-5.14)	
			Dominant model		
	AA	149 (55.6)	80 (52.6)	1	0.027

AGTR1	AC+CC	119 (44.4)	72 (47.4)	0.48 (0.24-0.92)	
	Recessive model				
	AA+AC	248 (92.5)	141 (92.8)	1	0.336
	CC	20 (7.5)	11 (7.2)	1.89 (0.52-7.06)	
	Overdominant model				
	AA+CC	169 (63.1)	91 (59.9)	1	0.006
	AC	99 (36.9)	61 (40.1)	0.38 (0.19-0.76)	

*Statistical significance tested by the likelihood ratio test, adjusted for age, sex, BMI, smoking, diabetes mellitus and hypertension.

Supplementary Table S2. Pairwise gene variants interactions and the risk of HF of NYHA class I/II assuming different models.

rs 1	rs 2	Model OR (95% CI), p*					
		**DOM**	**REC**	**HOM1-HET**	**HOM2-HET**	**HET-HOM1**	**HET-HOM2**
ACE	*AGTR1*	0.41 (0.20-0.82) 0.011	1.21 (0.12-12.50) 0.867	DD-AC 0.49 (0.15-1.67) 0.250	II-AC 1.49 (0.44-5.22) 0.526	ID-CC 3.60 (0.59-2.28) 0.168	ID-AA 1.66 (0.78-3.64) 0.192
ACE	*AGT*	1.30 (0.65-2.58) 0.458	0.77 (0.23-2.75) 0.684	DD-AG 1.03 (0.38-2.92) 0.949	II-AG 2.22 (0.70-7.90) 0.171	ID-GG 0.74 (0.31-1.82) 0.509	ID-AA 0.14 (0.04-0.51) 0.003
AGTR1	*AGT*	0.89 (0.45-1.75) 0.732	8.58 (0.23-728.8) 0.320	CC-AG 6.48 (1.10-4.44) 0.039	AA-AG 2.82 (1.32-6.26) 0.007	AC-GG 0.76 (0.31-1.90) 0.552	AC-AA 0.21 (0.05-0.85) 0.030

*FDR-adjusted p values for each pair of gene variants separately

Supplementary Table S3. Association of the A(-6)G *AGT*, I/D *ACE* and A1166C *AGTR1* polymorphisms with heart failure in NYHA class III-IV patients in regard to codominant, dominant, recessive or overdominant model of inheritance.

Gene	Genotypes	HF patients	Controls	OR (95% CI)	p*
AGT			Codominant model		
	AA	37 (25.0)	37 (29.6)	1	0.227
	AG	74 (50.0)	55 (44.0)	2.07 (0.80-5.56)	
	GG	37 (25.0)	33 (26.4)	1.10 (0.38-3.22)	
			Dominant model		
	AA	37 (25.0)	37 (29.6)	1	0.275
	AG+GG	111 (75.0)	88 (70.4)	1.64 (0.68-4.04)	
			Recessive model		
	AA+AG	111 (75.0)	92 (73.6)	1	0.396
	GG	37 (25.0)	33 (26.4)	0.69 (0.29-1.63)	
			Overdominant model		
	AA+GG	74 (50.0)	70 (56.0)	1	0.087
	AG	74 (50.0)	55 (44.0)	1.97 (0.91-4.42)	
ACE			Codominant model		
	II	24 (16.2)	36 (23.7)	1	0.565
	ID	84 (56.8)	81 (53.3)	0.66 (0.22-1.91)	
	DD	40 (27.0)	35 (23.0)	0.52 (0.15-1.73)	
			Dominant model		
	II	24 (16.2)	36 (23.7)	1	0.349
	ID+DD	124 (83.8)	116 (76.3)	0.61 (0.22-1.72)	
			Recessive model		
	II+ID	108 (73.0)	117 (77.0)	1	0.462
	DD	40 (27.0)	35 (23.0)	0.72 (0.30-1.71)	
			Overdominant model		
	II+DD	64 (43.2)	71 (46.7)	1	0.966
	ID	84 (56.8)	81 (53.3)	0.98 (0.45-2.12)	
AGTR1			Codominant model		
	AA	77 (52.0)	80 (52.6)	1	0.329
	AC	62 (41.9)	61 (40.1)	0.55 (0.24-1.21)	
	CC	9 (6.1)	11 (7.2)	0.76 (0.16-3.68)	
			Dominant model		
	AA	77 (52.0)	80 (52.6)	1	0.152
	AC+CC	71 (48.0)	72 (47.4)	0.57 (0.26-1.23)	
			Recessive model		
	AA+AC	139 (93.9)	141 (92.8)	1	0.982
	CC	9 (6.1)	11 (7.2)	1.02 (0.23-4.64)	
			Overdominant model		
	AA+CC	86 (58.1)	91 (59.9)	1	0.146
	AC	62 (41.9)	61 (40.1)	0.57 (0.26-1.22)	

*Statistical significance tested by the likelihood ratio test, adjusted for age, sex, BMI, smoking, diabetes mellitus and hypertension.

Supplementary Table S4. Pairwise gene variants interactions and the risk of HF of NYHA class III/IV assuming different models.

rs 1	rs 2	Model OR (95% CI), p*					
		**DOM**	**REC**	**HOM1-HET**	**HOM2-HET**	**HET-HOM1**	**HET-HOM2**
ACE	*AGTR1*	0.41 (0.18-0.91) 0.023	1.00 (0.09-13.79) 0.997	DD-AC 0.91 (0.25-3.38) 0.890	II-AC 3.22 (0.76-13.13) 0.110	ID-CC 1.35 (0.20-11.95) 0.770	ID-AA 2.85 (1.18-7.29) 0.020
ACE	*AGT*	1.13 (0.51-2.49) 0.762	0.34 (0.08-1.69) 0.184	DD-AG 1.24 (0.41-3.87) 0.702	II-AG 1.71 (0.35-7.82) 0.499	ID-GG 0.71 (0.25-2.00) 0.513	ID-AA 0.61 (0.19-1.93) 0.396
AGTR1	*AGT*	0.89 (0.40-1.95) 0.769	11.19 (0.16-1271.52) 0.336	CC-AG 2.60 (0.33-26.23) 0.377	AA-AG 1.83 (0.73-4.66) 0.198	AC-GG 0.36 (0.11-1.12) 0.077	AC-AA 0.60 (0.15-2.55) 0.488

*FDR-adjusted p values for each pair of gene variants separately
